# Optimization of breeding program design through stochastic simulation with evolutionary algorithms

**DOI:** 10.1093/g3journal/jkae248

**Published:** 2024-11-04

**Authors:** Azadeh Hassanpour, Johannes Geibel, Henner Simianer, Antje Rohde, Torsten Pook

**Affiliations:** Department of Animal Sciences, Animal Breeding and Genetics Group, University of Goettingen, Albrecht-Thaer-Weg 3, Goettingen 37075, Germany; Center for Integrated Breeding Research, University of Goettingen, Carl-Sprengel-Weg 1, Goettingen 37075, Germany; Department of Animal Sciences, Animal Breeding and Genetics Group, University of Goettingen, Albrecht-Thaer-Weg 3, Goettingen 37075, Germany; Center for Integrated Breeding Research, University of Goettingen, Carl-Sprengel-Weg 1, Goettingen 37075, Germany; Institute of Farm Animal Genetics, Friedrich-Loeffler-Institut, Neustadt 31535, Germany; Department of Animal Sciences, Animal Breeding and Genetics Group, University of Goettingen, Albrecht-Thaer-Weg 3, Goettingen 37075, Germany; Center for Integrated Breeding Research, University of Goettingen, Carl-Sprengel-Weg 1, Goettingen 37075, Germany; Coordination Center – Innovation Center Gent, BASF Belgium, Ghent 9052, Belgium; Department of Animal Sciences, Animal Breeding and Genetics Group, University of Goettingen, Albrecht-Thaer-Weg 3, Goettingen 37075, Germany; Center for Integrated Breeding Research, University of Goettingen, Carl-Sprengel-Weg 1, Goettingen 37075, Germany; Animal Breeding and Genomics, Wageningen University & Research, P.O. Box 338, Wageningen, AH 6700 Netherlands

**Keywords:** optimization, evolutionary algorithm, resource allocation, kernel regression, breeding program, stochastic simulation

## Abstract

The effective planning and allocation of resources in modern breeding programs is a complex task. Breeding program design and operational management have a major impact on the success of a breeding program and changing parameters such as the number of selected/phenotyped/genotyped individuals in the breeding program will impact genetic gain, genetic diversity, and costs. As a result, careful assessment and balancing of design parameters is crucial, taking into account the trade-offs between different breeding goals and associated costs. In a previous study, we optimized the resource allocation strategy in a dairy cattle breeding scheme via the combination of stochastic simulations and kernel regression, aiming to maximize a target function containing genetic gain and the inbreeding rate under a given budget. However, the high number of simulations required when using the proposed kernel regression method to optimize a breeding program with many parameters weakens the effectiveness of such a method. In this work, we are proposing an optimization framework that builds on the concepts of kernel regression but additionally makes use of an evolutionary algorithm to allow for a more effective and general optimization. The key idea is to consider a set of potential parameter settings of the breeding program, evaluate their performance based on stochastic simulations, and use these outputs to derive new parameter settings to test in an iterative procedure. The evolutionary algorithm was implemented in a Snakemake workflow management system to allow for efficient scaling on large distributed computing platforms. The algorithm achieved stabilization around the same optimum with a massively reduced number of simulations. Thereby, the incorporation of class variables and accounting for a higher number of parameters in the optimization framework leads to substantially reduced computing time and better scaling for the desired optimization of a breeding program.

## Teaser

The optimization of a breeding program is a highly complex task with various design options involved. Hassanpour et al. have developed an evolutionary algorithm optimization method to address the resource allocation problems in breeding programs. Evolutionary algorithm involves creating a population of potential solutions and iteratively evolving them over time using different operators. The optimization process with thousands of individual simulations and hundreds of iterations is implemented via the Snakemake workflow to allow for reproducible, flexible, and scalable management of computing dependencies.

## Introduction

With the rise of genomics, advancements in biotechnology, statistical modeling, and shifting market demands, breeding programs have transformed substantially in recent decades. While these advancements help breeders refine their strategies, they also turn modern breeding programs into complex resource allocation problems, requiring a balance between short-term genetic gain and long-term sustainability (Henryon *et al.* 2014; Berry 2015; Hickey *et al.* 2017; Simianer *et al.* 2021). Breeding programs require significant resources and time, with the outcomes of a certain decision often becoming apparent only after several years. As breeding actions are interdependent, where changes in one step affect key characteristics like genetic gain, diversity, and cost simultaneously, breeders must consider risks and the trade-offs of each decision, carefully evaluating the costs and benefits of every potential allocation of resources (Harris *et al.* 1984; Mi *et al.* 2014; Simianer *et al.* 2021; Jannink *et al.* 2024). A strategy that recently gained in popularity is to use stochastic simulation to assess breeding program design before practically implementing them (Henryon *et al.* 2014; Covarrubias-Pazaran *et al.* 2021). For this, a variety of software can be used, including MoBPS (Pook *et al.* 2020), AlphaSim (Faux *et al.* 2016; Gaynor *et al.* 2020), Adam (Liu *et al.* 2018), and QMsim (Sargolzaei and Schenkel 2009).

However, the optimization of a breeding program design using stochastic simulation is complicated by the fact that the output of a simulation is only the realization of a stochastic process. Thus, multiple replicates are necessary to reliably estimate the expected outcomes of a breeding scheme (Bančič *et al.* 2024). Since breeding programs often involve numerous parameters, it is not feasible to simulate all possible breeding designs many times, as simulating a real-world breeding scheme can be computationally expensive. Therefore, analysis of breeding program designs using stochastic simulations is usually limited to a few potentially interesting scenarios and research studies focusing on very specific aspects of breeding design (Lorenz 2013; Henryon *et al.* 2014; Hickey *et al.* 2014; Woolliams *et al.* 2015; Gorjanc and Hickey 2018; Moeinizade *et al.* 2019, 2022; Wellmann 2019; Allier *et al.* 2020; Büttgen *et al.* 2020; Duenk *et al.* 2021; Ojeda-Marín *et al.* 2021).

Recently, we introduced a framework for optimizing breeding program designs to address and generalize different aspects of the breeding program more effectively (Hassanpour *et al.* 2023). In that study, the primary focus was not placed on employing specific breeding actions, instead, the emphasis was on providing a general optimization framework that facilitates the simultaneous optimization of multiple design parameters within breeding schemes that significantly influence the efficiency of the program. These factors include the definition of breeding objectives, the available budget, and the number of individuals in each step of the breeding program, along with other relevant considerations.

The framework we suggested in Hassanpour *et al.* (2023) utilizes stochastic simulation to evaluate the outcomes of the breeding program and subsequently assess the scheme on an objective function/breeding goal. In the process of optimizing a breeding program, one begins with a random search algorithm to explore disparate areas of the search space to examine a broad range of parameter values and obtain a preliminary set of different breeding programs for optimization. Subsequently, kernel regression is employed by fitting a local regression curve to the data points, which are essentially a weighted average in which more similar breeding schemes are weighted stronger. Kernel regression smooths the results derived from the initial stage to filter out the noise and create a more discernible understanding of the potential regions where optimal solutions may be found. Following the application of kernel regression, the smoothed data provide an indication of the prospective ‘optima’ within the search space. The search is then concentrated on these narrowed-down regions, resulting in a substantial reduction of the overall search space. Finally, this entire process is performed iteratively with each successive iteration using the refined results of the previous round.

While this approach has proven effective in improving optimization results, its application is constrained to optimizing only a limited number of parameters. As the number of parameters for optimization increases, the computational demands for performing a sufficient number of simulations to obtain a broad coverage of the search space increases exponentially (Härdle and Müller 1997; Gutjahr and Pichler 2016). Additionally, we faced a challenge with a procedure that required manually narrowing down the search space through iterative steps and visual inspection. Recognizing these challenges, there is a need to create a new optimization framework that requires fewer simulations and is fully automated.

Traditional optimization techniques such as the steepest descent method, conjugate gradient method, and quasi-Newton method (Kiefer and Wolfowitz 1952; Back *et al.* 2008; Burke *et al.* 2020) tend to struggle in the settings of breeding planning with high dimensional search spaces and stochasticity in the evaluation of an objective function. Stochastic optimization techniques (Fouskakis and Draper 2002) provide a framework to cope with exactly these challenges and include techniques such as genetic and evolutionary algorithm (Pierreval and Tautou 1997; Alberto *et al.* 2002), simulated annealing (Ahmed *et al.* 1997), and Bayesian optimization (Schonlau 1998).

Previous research in the field of breeding planning is however limited to the application of Bayesian optimization in simplified settings with a fixed budget and only use of continuous variables (Diot and Iwata 2022; Jannink *et al.* 2024). A further issue for the optimization is that the evaluation of the objective function is computationally very expensive. Therefore, the chosen optimization technique should allow several evaluations to be carried out in parallel.

When optimizing complex problems with a high number of parameters and large search spaces, evolutionary algorithms (EAs) have gained popularity due to their ability to address the mathematical complexities inherent to real-world optimization problems, i.e. mixed class and continuous decisions, multiple objectives, uncertainty, computationally demanding simulations, etc. (Holland 1992; Bäck *et al.* 2000; Deb 2001; Michalewicz and Fogel 2004; Sivanandam and Deepa 2008; Eiben and Smith 2015; Katoch *et al.* 2021; Jeavons 2022).

In EAs, the terminology is borrowed from breeding, but it should not be confused with biological concepts. To ensure clarity, we define the following terms used solely within the context of the EA and do not refer to breeding terminology throughout this manuscript. “Parameter settings” refers to the variations in the design parameters of a breeding scheme that are subject to optimization. The “population” refers to the set of parameter settings. Each parameter setting in the population is evaluated using an “objective function,” which assigns a score based on the outcomes of the simulations, indicating how well a hypothetical breeding program would perform. The best parameter settings, or “parents,” are “selected,” producing new parameter settings (“offspring”) through different operators in each iteration. These operators combine and modify the selected best parameter settings to form new parameter settings, often involving “recombination” (combining promising parameter settings) and “mutation” (minor modification of a parameter settings).

Although many EAs exist, they are often problem-specific (Slowik and Kwasnicka 2020). Depending on the nature, complexity, and dimensionality of the problem, different variations of recombination and mutation operators are employed for optimization (Sipper *et al.* 2018). Designing effective operators is a crucial aspect of EA development, as they directly impact the algorithm’s ability.

For this reason, in this study, we introduce a novel EA framework specifically designed to optimize the design parameters of breeding programs, using a dairy cattle breeding program as an example suggested in Hassanpour *et al.* (2023). Our proposed framework can optimize both continuous and class design parameters and is adaptable for any breeding scenario, regardless of the species, methodology, resources, or genetic traits involved, providing a versatile tool for a variety of breeding objectives and therefore applies to any plant or animal breeding program as long as it can be simulated/evaluated via stochastic simulations.

## Materials and methods

**Fig. 1.**
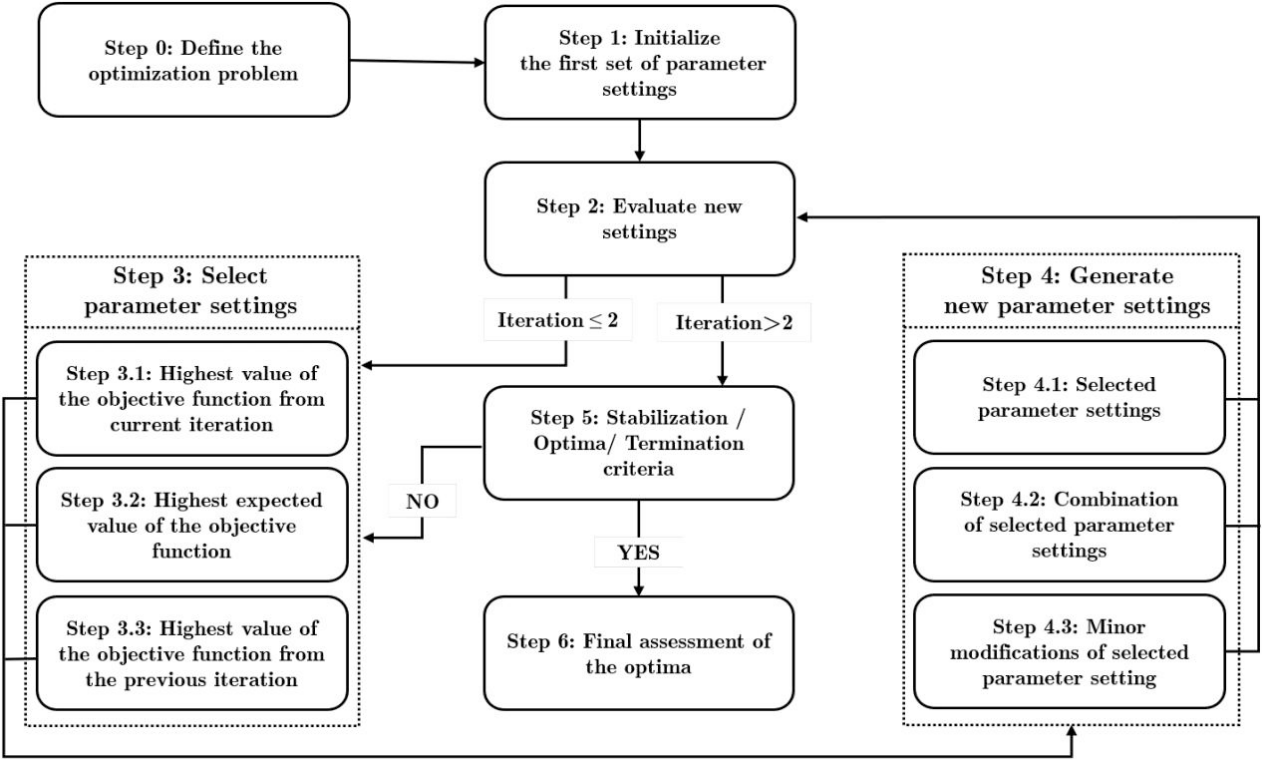
Procedure proposed for optimization via evolutionary algorithm.

In this study, we introduce a comprehensive framework for optimizing breeding scheme designs using an EA. The EA is structured as an iterative process, wherein steps 2 to 5 are reiterated until a termination criterion is met. For illustrative purposes, we will outline the individual steps of the algorithm using the same dairy cattle breeding program previously examined in Hassanpour *et al.* (2023). Figure 1 provides a schematic summary of the overall steps of our EA framework, representing key steps and their interconnections within the optimization process.

### Step 0: Definition of the optimization problem

Firstly, the breeding problem is formulated as an optimization problem, defining objectives, constraints, and decision variables. Two main types of decision variables are considered: class variables with a limited number of possible realizations (e.g. whether genomic selection is applied in a specific step of the breeding program) and continuous variables that can take values from a continuous scale, or at least a large number of discrete realizations. (e.g. number of candidates selected, phenotyped, and genotyped, or weights in a selection index).

Below, we present scenario 1, which serves as an example to illustrate the formulation of a breeding program design for an optimization problem as a baseline scenario. Subsequently, we showcase three additional examples to highlight the versatility of our EA framework, each representing a modification of scenario 1. These alternative scenarios are detailed towards the end of this section.

#### Scenario 1—Traditional dairy cattle scheme

**Fig. 2.**
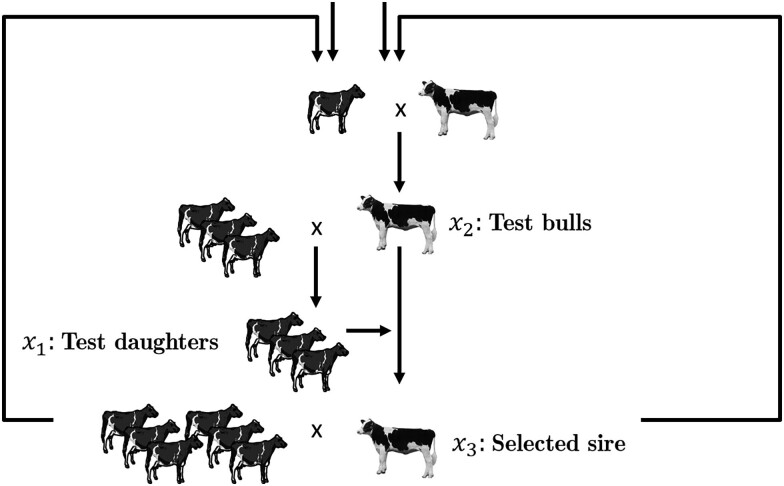
A dairy cattle breeding scheme.

Here, we are considering a traditional dairy cattle scheme, illustrated schematically in Fig. 2 as suggested in our previous study Hassanpour *et al.* (2023). As performance traits of bulls cannot be directly measured, preselected test bulls ($x_{2}$) must be mated with cows to produce test daughters ($x_{1}$). Bulls are selected as sires ($x_{3}$) based on the performance of their offspring. For simplification purposes, we are explicitly not considering the genotyping as commonly done in dairy cattle breeding in the last 15 years (VanRaden *et al.* 2009), and both bulls and cows are chosen based on pedigree breeding value estimates. We are considering here only a single quantitative trait (milk yield), with heritability ($h^{2}$) of 0.30.

The three parameters we consider for optimization are:

1.
$x_{1}$ : number of test daughters
2.
$x_{2}\;:\;$ number of test bulls
3.
$x_{3}$ : number of selected sires

As constraints, the breeding program at hand is limited by an annual budget of 10,000,000 Euros with housing costs of 3,000 Euros per bull and 4,000 Euros per cow.


$$\begin{array}{rl} & x_{1}+x_{2}+x_{3}\geq 0\\ & 4,000x_{1}+3,000x_{2}-10,000,000\leq 0 \end{array}$$

Note that, the initial bounds established during the initialization step are not constraints that are enforced throughout the optimization process. Instead, they serve as a starting point, and the variables may deviate from these bounds as the optimization algorithm iterates and refines the solution.

Exactly as in our previous study (Hassanpour *et al.* 2023), the objective function (*m*) is a linear combination of the expected genetic gain (*g*) and the expected inbreeding level (*f*) after 10 generations (5 years of burn-in + 10 years of future breeding), to prioritize/weigh between the genetic gain and diversity:


m(x)=g(x)−50×f(x).

### Step 1: Initialize the first set of parameter settings

To initialize the evolutionary framework, it is necessary to generate a starting population of potential breeding program designs to consider. For this, we generally propose to use a generalized Bernoulli distribution for class variables and a uniform distribution for continuous variables. It should however be noted that depending on constraints a more sophisticated sampling procedure might be required, especially when the variables are interdependent, and logical dependencies for each decision may come from other decisions that share the same resources. For instance, if the maximum stable capacity is 3,000, allowing 500–1,000 males and 500–2,500 females, and 1,000 males are selected during the sampling, the number of females cannot exceed 2,000 to fulfill the overall stable capacity of 3,000. This adjustment should be applied during the first step of sampling and throughout the process of optimization.

As spending more budget is not penalized in the target function, we know a priori that an optima will make use of the full budget, Hence, a scaling step is applied to variables $x_{1}$ and $x_{2}$ to meet the budget constraint. For example, if $x_{1}=1,025$, $x_{2}=300$, and the total cost is 5,000,000 Euros, both values are doubled to match the budget of 10,000,000. If the budget cannot be exactly met due to rounding, the program cost is kept slightly below the maximum by rounding down and then increasing parameters as much as possible. To avoid unnecessary computations, unreasonable breeding program designs like the use of too many bulls or selecting just a single bull per year are excluded by additional constraints. These constraints are only enforced in the initialization step:


$$\begin{array}{rl} 100 & \leq x_{2}\leq 700\\ 3 & \leq x_{3}\leq 30. \end{array}$$

### Step 2: Evaluate new settings

In the next step, the suitability of all 600 breeding program designs needs to be evaluated regarding the breeding objective as given by the objective function *m*. For this evaluation, each respective breeding program is simulated via stochastic simulation. We here use the R package MoBPS (Pook *et al.* 2020) with scripts given in https://github.com/AHassanpour88/Evolutionary_Snakemake/tree/main/scripts, but other simulation tools can be integrated seamlessly by the user.

### Step 3: Select parameter settings

Based on the results from the previous step, we want to identify the most promising areas of the search space to investigate further in later steps. For this, we select the most promising parameter settings to be uses as “parents” for the next iteration, considering three strategies. The number of selected parents based on each step varies based on the iterations of the EA with values given below for iteration 11 onwards (see Table 1). In these iterations, 30 out of 300 parameter settings are selected.

**Table 1.** Number of parameter settings selected as parents (step 3) and generated settings (step 4) during the EA optimization process.

Iteration	Selected settings			Generated new settings		
	Step 3.1	Step 3.2	Step 3.3	Step 4.1	Step 4.2	Step 4.3
2–3	70	30	0	100	200	0
4–10	30	15	5	50	170	80
11–40	20	7	3	30	180	90

Note: Step 3.1 presents individual simulations with the highest objective function value from the current iteration, step 3.2 presents individual simulations with the highest expected value of the objective function determined by kernel regression, and step 3.3 presents individual simulations with the highest objective function value from the previous iteration and previous optima. Step 4.1 represents the sum of steps 3.1, 3.2, and 3.3. Step 4.2 represents the number of new settings (new breeding program designs) generated through a combination of selected parameter settings. Step 4.3 represents the number of new parameter settings created through minor modifications of selected settings.

####  

##### Step 3.1: Highest value of the objective function

In the first sub-step, 20 of the 30 most promising parameter settings are identified (“selected”) based on the value of the objective function that was derived solely based on the simulation of the parameter settings itself. This guarantees that the best-performing parameter settings are prioritized in the reproduction process, subsequently enhancing the likelihood of generating successful parameter settings.

##### Step 3.2: Highest expected value of the objective function

In the second sub-step, seven parameter settings are identified based on the highest expected value of the objective function. For this, we are employing the kernel regression method suggested in Hassanpour *et al.* (2023). By this, instead of evaluating each parameter setting individually, kernel regression computes a weighted average of performance values for multiple parameter settings. In contrast to step 3.1, this approach avoids bias towards region with more parameter settings tested overall. In contrast to Hassanpour *et al.* (2023), we are here proposing the use of an adaptive bandwidth by using the empirical standard deviation in the individual parameter in the current iteration.

##### Step 3.3: Highest value of the objective function from the previous iteration

In the third sub-step, three parameter settings from previous iterations are used. This typically refers to the best-performing parameter settings from the second-to-last iteration; however, if the expected performance of any previously suggested optima from any iteration based on kernel regression (see step 5) is higher than all parameter settings of the current iteration, these are added instead. Hereby, the risk of discarding potentially promising parameter settings due to stochasticity is reduced.

#### Management of parameter setting variability/heterogeneity/value distribution

We implement a strategy to manage variation within the EA, ensuring that highly similar parameter settings are not selected. For additional details on this approach, refer to Supplementary File S1.

### Step 4: Generate new parameter settings

Subsequently, the previously selected parameter settings are used to generate a set of new parameter settings that are evaluated in the next iteration. This process involves applying various techniques to create a new set of parameter settings, drawing inspiration from the process of meiosis. In the next step, we will discuss these criteria and how they contribute to the overall success of the evolutionary process. The number of generated settings will again vary based on which iteration the algorithm is in (see Table 1). The values given below are for iterations 11 onwards with a total of 300 settings generated.

#### Step 4.1: Selected parameter settings

In the first sub-step, all 30 previously chosen parameter settings are considered again in the next iteration. This criterion facilitates assessing the same parameter settings using a new random seed in each iteration to reduce variance, resulting in a more robust evaluation of their performance.

#### Step 4.2: Combination of selected parameter settings

In the second sub-step, 180 parameter settings are generated by combining two randomly chosen parental parameter settings in step 3, denoted as $X=(x_{1},x_{2},\ldots )$ and $Y=(y_{1},y_{2},\ldots )$. The goal of this step is to fine-tune and explore the areas between two potential parameter settings. By using these parents, a new parameter setting $Z=(z_{1},z_{2},\ldots )$ is created in each case.

For continuous variables, this is done by the use of a weighted average:


$$z_{i}\;=wx_{i}+(1-w)y_{i}\;\text{with}\;w∼U(0,1).$$

For class variables, we randomly sample $z_{i}$ with an equal probability of belonging to either the class of $x_{i}$ or $y_{i}$. In our minimal example, it is necessary to furthermore round and scale continuous variables to obtain integer numbers while fulfilling the budget constraint (see step 1).

Following the combination process, we introduce small changes to the combined parameter settings, inspired by the process of allelic mutation in meiosis. For continuous parameters, the size of the mutation $t_{i}$ in parameter *i* is sampled from a uniform distribution with the range determined by the variance of the parameter and a mutation occurring in each respective parameter with a probability of $p_{\mathrm{activ},i}$:


$$\begin{array}{rl} Z_{mut} & =(z_{1}+m_{\mathrm{activ},1}t_{1},z_{2}+m_{\mathrm{activ},2}t_{2},\ldots )\\ & \;\text{with}\;t_{i}∼U(-2\sigma_{x_{i}},2\sigma_{x_{i}})\\ & \;\text{and}\;m_{\mathrm{activ},i}∼B(0,p_{\mathrm{activ},i}) \end{array}$$

here $\sigma_{x_{i}}$ denotes the standard deviation and is derived based on the empirical variance of the parameter settings of the current iterations. In our algorithm, in the first iteration, $p_{\mathrm{activ},i}$ is set to 0.2, indicating a 20% chance of mutation for all parameters.

As the algorithm progresses, continuous decision variables are fine-tuned for specific class variables. To avoid the generation of new parameter settings with little promise, the likelihood of combining settings with different class variables is reduced. By iteration 2, this probability is reduced by 20% and continues to decrease by 10% per iteration, reaching an 80% reduction. Similarly, the mutation rate for class variables ($p_{\mathrm{activ},i}$) is reduced by 10% in iteration 2, and then by 5% per iteration until a reduction of 40% is reached.

#### Step 4.3: Minor modifications of selected parameter setting

In the third sub-step, we are refining all parameter settings that have already been selected, either from step 4.1 or step 4.2 by making additional small changes/mutations to them (see step 4.2). These modifications not only fine-tune the current solutions but also allow exploration beyond the regions currently being investigated, helping to discover new, potentially promising areas of the parameter space. To avoid generating a setting already generated in step 4.1, mutation rates $p_{\mathrm{activ},i}$ are increased to 0.3 and the sampling procedure is repeated in case no mutations are performed.

As a refinement to step 4, mutation rates can be adjusted based on the observed changes in each parameter during previous iterations. For example, a binary parameter that consistently shows superior results with one of the parameter settings should undergo less frequent mutation. For details on the approach used in our minimal example, the interested reader is referred to Supplementary File S2.

### Step 5: Stabilization/optima/termination criteria

To derive the optima, we suggest to first employing a kernel density estimation to determine which areas of the search space include sufficient coverage to reliably assess them. We here propose to only consider those settings from the last five iterations with a value for the kernel density estimation above the 20% quantile of these parameter settings, to avoid using parameter settings in sparsely sampled areas. For the estimation of the kernel density estimation, only simulations from the last five iterations are used.


$$f(y)=\frac{1}{n^{p}\cdot h_{1}\cdots h_{p}}\sum_{i}K\left(\frac{y_{1}-x_{i}{,}_{1}}{h_{1}},\ldots ,\frac{y_{n}-x_{i}{,}_{p}}{h_{p}}\right)$$

with *p* being the number of decision variables and using the empirical standard deviation in the last five iterations as the bandwidth $h_{j}$ and the use of a multivariate Gaussian kernel for *K*:


$$K(y_{1},\ldots ,y_{p})=K_{1}(y_{1})\cdots K_{p}(y_{p})$$

with


$$K_{i}(x)=\frac{1}{\sqrt{2\pi }}\exp\;\left(-\frac{x^{2}}{2}\right)$$

Subsequently, the expected performance of all remaining parameter settings is estimated using a kernel regression (see step 3.2 in Hassanpour *et al.* 2023) using all simulations. The parameter setting with the highest value based on the kernel regression is used as the optima.

In our example, we performed 40 iterations without any termination criteria accessed.

### Step 6: Final assessment of the optima

After the optimum is identified, the finally obtained breeding scheme is analyzed in-depth, as the kernel regression will naturally be biased in an extrema (Härdle 1990). For this, the suggested parameter setting is simulated a high number of times, e.g. in our case 100 replicates, to get an unbiased estimate for expected performance but also assess variances.

### Demonstrating alternative examples to scenario 1

#### Scenario 2—Reduced initial search space

In scenario 2, we consider the same breeding program and resource allocation problem as in scenario 1, so the optima should be the same. Only difference between the scenarios is that we use an initial search space (step 1) that excludes the optimal parameter settings (2,368, 175, 19) identified in our previous study (Hassanpour *et al.* 2023). This setup allows us to assess the effectiveness of our EA algorithm in identifying the optimal parameters even when they are not part of the initial search space. The design parameters’ bounds are defined in the initialization (step 1) as follows:


$$\begin{array}{rl} 300 & \leq x_{2}\leq 500\\ 15 & \leq x_{3}\leq 25 \end{array}$$

#### Scenario 3—Addition of class variable

For showcasing the optimization of a class variable with two possible outcomes (yes/no, or 0/1), we extend scenario 1 by introducing a binary variable, $x_{4}$. This variable represents a breeding strategy that leads to improved phenotyping. For this study, we leave it open as to what this new breeding strategy is, but one could envision more uniform housing conditions, the use of electronic devices to measure physiological status, or large-scale collection of additional data like mid-infrared spectroscopy (Boichard and Brochard 2012; De Vries *et al.* 2015). This suggests that while the genetic variance remains constant, the residual variance decreases due to more precise measurements. Consequently, the heritability ($h^{2}$) increases from 0.30 to 0.32 when this binary variable is active.

Step 1 is accordingly adapted by sampling initial values for $x_{4}$ from a Bernoulli distribution $x_{4}∼B(0.5)$. We are here considering two versions of the scenario (3a/3b) with varying additional costs of 1,000€/10€ per phenotyped cow if the improved phenotyped is used ($x_{4}=1$), respectively. Resulting in a new budget constraints:


$$\begin{array}{rl} & 3a\;:\;x_{1}(4,000+1,000x_{4})+3,000x_{2}-10,000,000\leq 0\\ & 3b\;:\;x_{1}(4,000+10x_{4})+3,000x_{2}-10,000,000\leq 0 \end{array}$$

### Snakemake

The EA algorithm is iterative, involving multiple interdependent steps, where the computational demands for executing some steps are notably high, particularly the resource-intensive simulation of breeding programs in step 2. To effectively address this computational challenge, the implementation of parallel processing in an efficient manner is crucial.

**Fig. 3.**
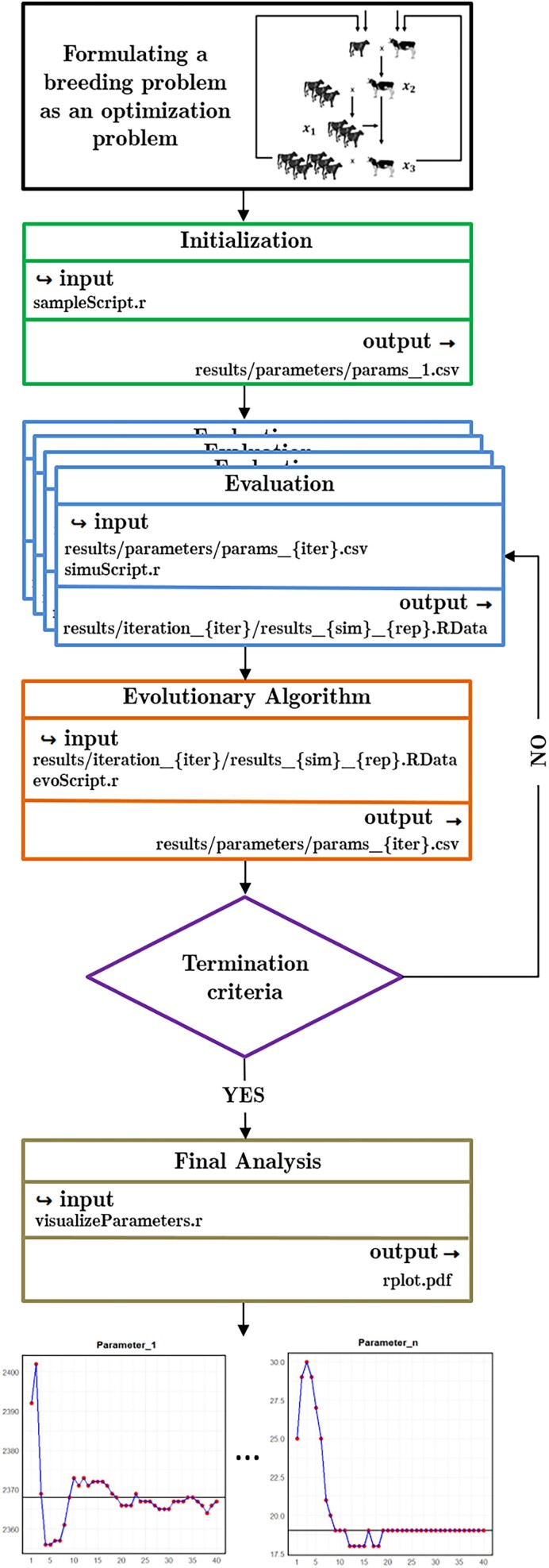
Example visualization of the Snakemake workflow. Shown are the rule names defined and their input–output relationships.

For this purpose, our optimization framework makes use of the automation provided by the Snakemake workflow management system (Mölder *et al.* 2021). An illustrative representation of the Snakemake workflow for our evolutionary optimization model is provided in Fig. 3. Our Snakemake process uses four rules, corresponding to the steps of the algorithm, which are initialization (step 1), evaluation (step 2), evolutionary algorithm (steps 3, 4, and 5), and the final in-depth analysis of the obtain optima (step 6).

To clarify, the individual simulations within step 2 are completely independent of each other and can easily be run in parallel using the built-in capabilities of Snakemake to seamlessly interact with various job scheduling systems (e.g. SLURM https://slurm.schedmd.com/documentation.html) on distributed hardware stacks, thus ensuring portability to a wide range of hardware setups. Those interested in configuring this setup can refer to the Snakemake plugin catalog at: https://snakemake.github.io/snakemake-plugin-catalog/index.html. This catalog provides a starting point for configuring Snakemake to work with various cluster schedulers, ensuring optimal distribution and execution of multiple tasks across the computing cluster.

### Computer hardware

All tests were executed on a server cluster with Intel Platinum 9,242 (2X48 core 2.3 GHz) CPUs using Snakemake toolkit version 7.21.0, which was configured to distribute single jobs via a SLURM scheduler to the backends of the cluster. Simulations were conducted on single nodes using a single core per simulation, taking approximately 15 minutes, and peak memory usage of 5 GB RAM per simulation. The computing time of all other steps combined increases approximately linearly in the number of iterations, but even in iteration 40 only took a negligible 7 s.

## Results

**Fig. 4.**
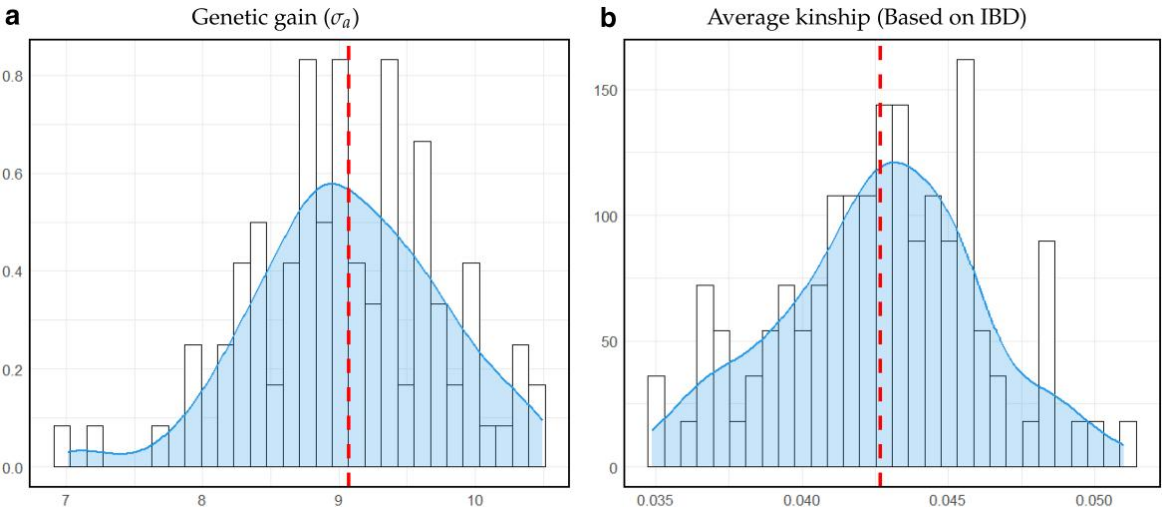
Realization of expected outcome for scenario 1 based on 100 replicates in a) for genetic gain ($\sigma_{a}$), b) for average kinship (based on IBD). The red dashed line represents the mean value, while the blue shaded area shows the probability density.

Application of our evolutionary framework to the optimization problem formulated in scenario 1 suggests a final optimum of 2,368 test daughters and 175 test bulls, of which 19 test bulls are selected ($x_{1}=2,368$, $x_{2}=175$, $x_{3}=19$) with an expected outcome for the target function of 107.041, with a genetic gain of 9.07 genetic standard deviations (Fig. 4a) and increase of the inbreeding level of 0.0426 (Fig. 4b) after 10 generations based on the averages of 100 replicates of this scenario.

**Fig. 5.**
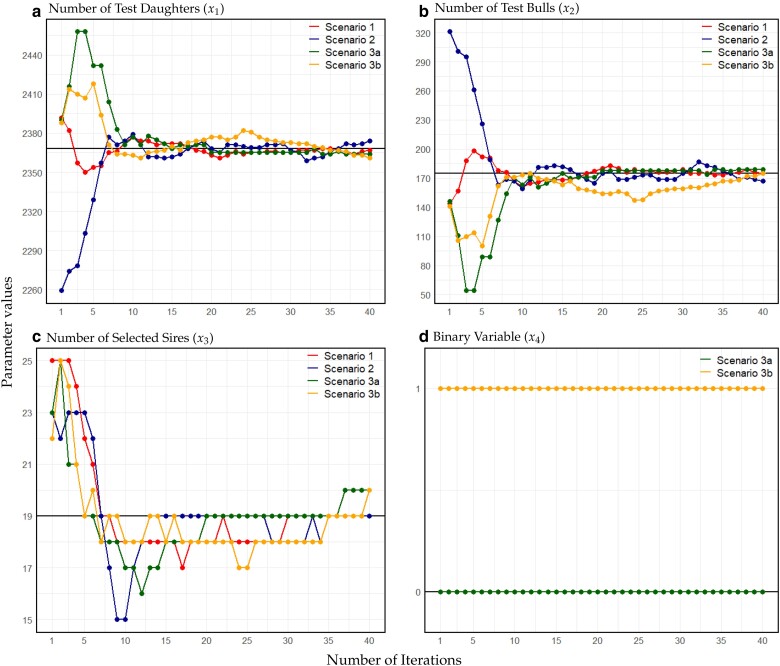
Suggested optima for the individual design parameters of the breeding program design for the number of test daughters a), test bulls b), and selected sires c), as well as the binary variable (d). The black horizontal line represents the estimated optima through comprehensive exploration, achieved by conducting over 100,000 simulations utilizing kernel regression (Hassanpour *et al.* 2023). a) Number of test daughters ($x_{1}$), b) number of test bulls ($x_{2}$), c) number of selected sires ($x_{3}$), and d) binary variable ($x_{4}$).

All three individual parameters very quickly reached values close to the finally suggested optima (Fig. 5a:5c). When evaluating the suggested optima per iteration based on all simulations conducted (to avoid effects of reduced bandwidth over iteration) even after seven iterations a value of 107.038 for the target function based on kernel regression was obtained (Fig. 4), despite kernel regression by design being downward biased in the optimum.

**Fig. 6.**
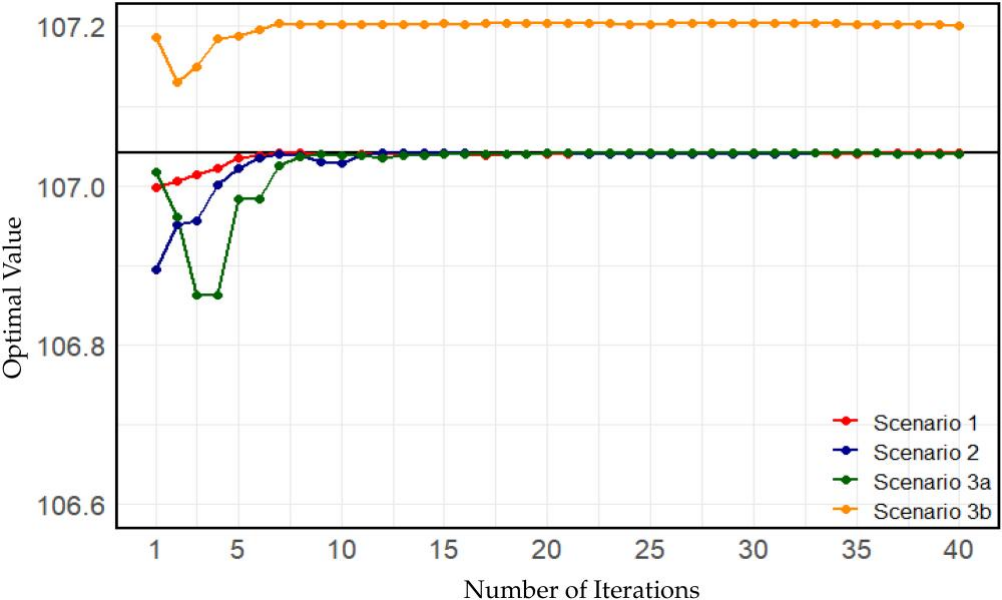
Performance of the suggested optima after each iteration assessed using kernel regression based on all simulations in scenario 1. The black horizontal line shows the estimated optima obtained from Hassanpour *et al.* (2023).

**Fig. 7.**
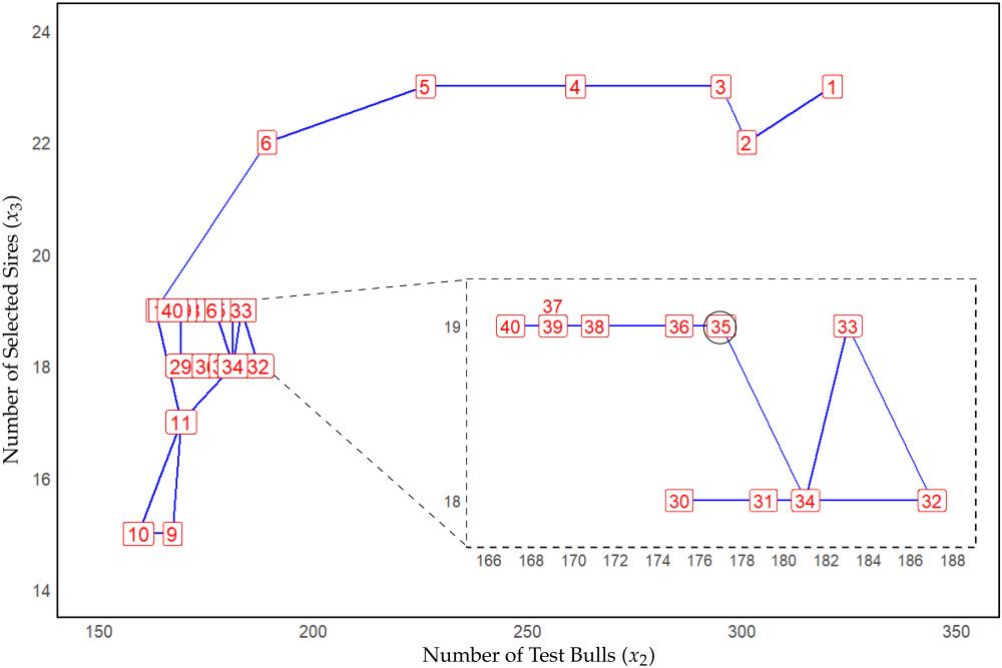
Suggested optima across iterations for scenario 2. Red labels denote iteration numbers, while the blue line illustrates the iterative pathway. The dashed segment zooms in on the overlapping iterations within the optimal range from iteration 30 to 40. The black circle denotes the reference point for optimal parameter settings obtained through kernel regression, involving over 100,000 simulations in our previous study (Hassanpour *et al.* 2023).

In scenario 2, we obtain an optimum very similar to those in scenario 1, with values $x_{1}=2,374$, $x_{2}=167$, and $x_{3}=19$ after 40 iterations. Even though the suggested optima in the first couple of iterations are performing slightly worse, the value for the target function is on par with the optima suggested in scenario 1 after seven iterations (Fig. 6). For the individual parameters, more change can be observed with the initial iterations suggesting using more test bulls, due to limitations in the initial search space. Similarly, a higher number of sires are selected ($x_{3}$) to use a similar selection intensity. In the first 10 iterations of scenario 2, the primary emphasis is on quickly bringing $x_{2}$ into its optimal range due to the higher overall impact of $x_{2}$ on the optimum. Overall, more change in the individual parameters is observed with $x_{3}$ in iteration 10 being as low as 15 (Fig. 5c). A detailed overview of the changes in $x_{2}$ and $x_{3}$ is given in Fig. 7.

**Fig. 8.**
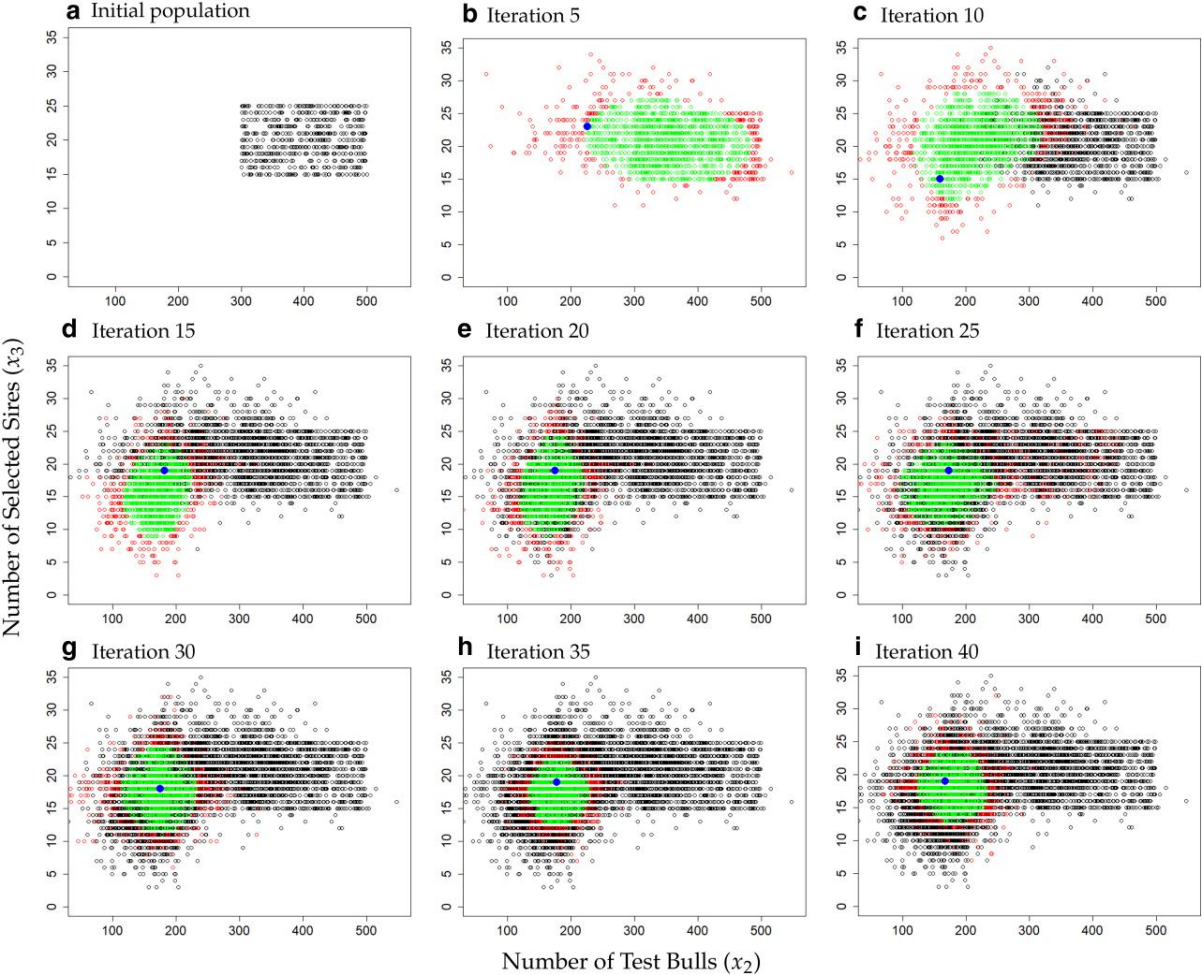
Estimates of the optimum values in scenario 2: a) initial population, b) iteration 5, c) iteration 10, d) iteration 15, e) iteration 20, f) iteration 25, g) iteration 30, h) iteration 35, and i) iteration 40. The black points in the illustration represent simulations from more than five iterations back that are not considered as potential optima. Red points represent simulations that were excluded as optima due a low kernel density estimation. Green points represent parameter setting optima from the kernel density estimation with the blue dot representing the finally chosen parameterization.

Although parameter settings with smaller $x_{2}$ values do exist in early iterations (Fig. 8b), these are not considered in deriving the optima (step 5) due to the low kernel density (red area). In later iterations, the area of solutions considered as the optima more and more shifts towards the area with the expected optima (green area, Fig. 8c:8i).

In scenario 3a, the optimum obtained is similar to those in scenarios 1 and 2, with the binary variable not being active (x=(2,365,179,20,0)). In terms of how fast the algorithm approaches a stable solution, more variation in individual parameters is observed in early iterations, particularly with iterations 4 and 5 suggesting optima that are later identified as poor solutions (Figs. 5a–c and 6) caused by the stochasticity in the evaluation of the target function (step 2).

In contrast, the binary is active in scenario 3b which allows for a higher overall value of the target function of 107.200 that represents a statistically significant improvement based on a t-test (Student 1908) (P<0.00663). Due to the higher overall housing costs, the number of cows and bulls is slightly decreased with a finally suggested optimum of x=(2361,175,20,1).

**Fig. 9.**
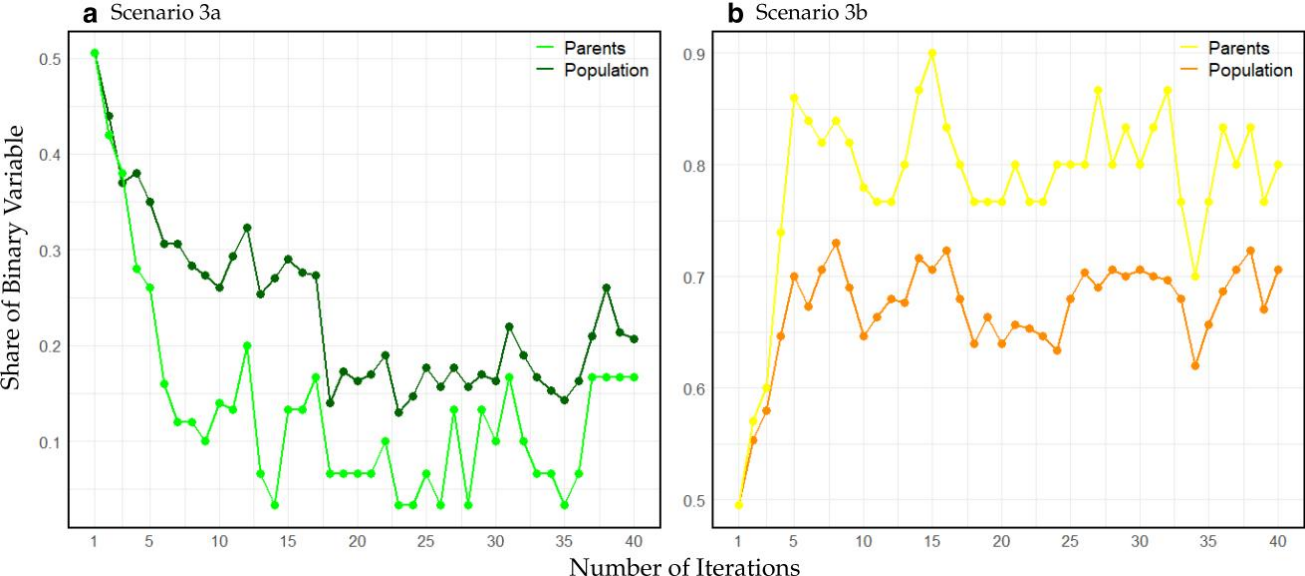
Share of binary variable to increase heritability of phenotyping being active in each iteration in a) for scenario 3a with green being the share of parents and darkgreen being the share of population, b) for scenario 3b with yellow being the share of parents and darkorange being the share of population.

Regarding the binary parameter, mutation rates in scenario 3a are reduced to half from iteration 17 onwards, while for scenario 3b mutation rates remain high for the entire 40 iterations. In both scenarios, the share of the favorable binary in the selected parameter settings (step 3) is higher than in the overall population but both cases are considered over the entirety of the simulations with 18%/25% of the parameter settings of the respective alternative binary setting (Fig. 9a and b).

## Discussion

In this study, we present a novel EA framework developed to optimize breeding program design with both class and continuous design variables that are suitable for the joint optimization of multiple design parameters of breeding programs in a computationally efficient matter. Given the dynamic nature of breeding programs, which constantly face new genetic information, environmental factors, technologies, and market demands, regular optimization of breeding programs is essential for maintaining efficiency. By continuously monitoring the program’s performance, areas for improvement can be identified, allowing for timely adjustments to prevent any negative effects on the overall effectiveness of the breeding program. Insights from the results highlight six key points for discussion:

### Evaluating the performance of the framework

The here-developed framework represents a much-enhanced version of the kernel regression pipeline suggested in Hassanpour *et al.* (2023). The iterative nature of the EA, where each iteration produces more data (simulations) for kernel regression allows for more reliable and efficient identification of suitable parameter settings for a breeding scheme, with the kernel regression still used as a core element to cope with the challenges of optimization problems that involve stochastic noise in the evaluation of the target function, a common issue when utilizing stochastic simulations (Hart and Belew 1996; Liang *et al.* 2000).

The developed framework provides a lot of flexibility to easily adapt parts of the algorithm to improve its efficiency but also the breeding program design itself. Nonetheless, models also provide robustness, as shown in scenarios 1, 2, and 3a with independent runs obtaining very similar final optima. Note here that the obtained optimum across the iterations of the EA can gradually change with decreasing bandwidth as the algorithm approaches a stable solution. Therefore, the suggested “optimum” will most likely not be the exact optimum but at least be very close to it and the suggested optimum in scenario 1 exactly matches the optimum from Hassanpour *et al.* (2023) should therefore mostly be seen as a coincidence. Note that the stochasticity in the evaluation of the target function will naturally cause minor deviations between runs and although differences in solutions will exist, practically, suggested optima between the three scenarios that should have the same optimum all were very similarly.

Assessing EAs’s performance in terms of speed and computational effort is broadly defined to include various sensible metrics, such as the number of iterations, CPU time, or more general the resulting financial cost for running the full pipeline (Eiben and Smit 2011), with the number of simulations required being the main driver of computational load in our framework. The efficacy of our EA framework in reducing computational resources is highlighted through its performance across all scenarios. For example, in scenario 1 only 2,400 simulations were required to achieve similar results as compared to our previous kernel regression approach which relied on more than 100,000 simulations (Hassanpour *et al.* 2023). This demonstrates a considerable reduction in computational load, with the algorithm achieving a more than 40-fold decrease compared to the kernel regression method.

Robustness is particularly highlighted by scenario 2, demonstrating that the EA algorithm can find optimal solutions even outside of the initial search space. This is an advantage over our previously established kernel regression method (Hassanpour *et al.* 2023), which relies on predefined parameter bounds and cannot dynamically adapt its search space. As such, it falls short in terms of automation and efficiency.

Jannink *et al.* (2024) reported high variability in the outcomes of different runs of the Bayesian optimization framework depending on small changes such as input genotypes and therefore lacking the ability to draw general conclusions from the obtained results. In our case, input genotypes and trait architecture were randomly sampled for each simulation. Very similar optima were obtained in the scenarios that should have the same optimum (scenarios 1, 2, and 3b), even with a less-than-ideal initial search space or when adding a binary decision variable. This serves as a strong indicator of the generality and stability of the approach. Additionally, scaling for a higher number of parameters should be greatly improved, as traditional kernel regression scales exponentially with the number of assessed parameters. This emphasizes the practical advantages of evolutionary algorithms in optimizing large-scale breeding program designs.

In this study, we examined the implementation of an evolutionary algorithm within a simplified breeding scheme to maintain focus on the method itself. The applicability of our approach, as well as the practical advantages of evolutionary algorithms in optimizing complex, large-scale breeding program designs involving up to 20 parameters for optimization, will be addressed in a companion study that is currently in preparation (Hassanpour *et al.* 2024).

### Class variables

In the process of optimizing breeding scheme design, Jannink *et al.* (2024) showed that there are difficulties when using Bayesian optimization to allocate budgets effectively in breeding schemes. One of the limitations faced in this investigation and our previous work (Hassanpour *et al.* 2023) is the lack of support for class variables. Our EA strategy helps addressing the computational intensity associated with continuous optimization problems involving class variables. To further test the robustness of our optimization algorithm, we intentionally introduced a binary decision variable into the problem formulation. Adding a binary decision variable to the optimization problem introduces an additional layer of complexity, making the search for the optimal solution more challenging, as it allows for the inclusion of less favorable options that the algorithm must evaluate. This was demonstrated in scenario 3a, where the search space became larger due to the inclusion of less desirable options with a high cost of phenotyping when $x_{4}=1$. However, despite this increased difficulty due to the wider search space created by less favorable parameter settings, the algorithm demonstrated robust performance and effectively converged to the same optimal solution as without the binary variable being active.

However, the use of class variables should be approached with caution. These problems can be computationally intensive not only due to their combinatorial nature but also due to the increase in the number of possible outcomes (Pelamatti *et al.* 2018). Particularly with a high number of class variables, the total number of combinations will rapidly increase to $k^{n}$ for *n* class variables that all can take *k* values. If possible, we would therefore strongly recommend using as low a number of class variables as possible, e.g. in the here-considered scenario 3b the additional cost of improved phenotyping was extremely low which from a human side makes it quite obvious to spend this additional money. However, as the resulting improvement for the target function is low, the overall upside is low, and high overall stochasticity in the evaluation is observed, the EA for all 40 iterations considered both binary settings. On the contrary, more substantial differences, such as an increase in the costs of phenotyping of 1,000 Euro for a marginal improvement in precision, are more easily detectable by the algorithm to be unsuitable. Therefore, we recommend that if such a design decision seems straightforward based on quantitative genetic theory or intuition, it might be advisable to simplify the optimization process by fixating such a class variable from the beginning or running separate optimization frameworks for both binary settings.

### Modified parameter settings

Within our suggest EA framework a design choices (e.g. mutation rates, how many parameter settings to simulate etc.) are made. Although some of these choices seem arbitrary, most of these choices were extensively evaluated by use for optimization with much simplified evaluation of the target function (to save computing time) and a known optimum. The presented design choice in our method section represent settings that should represent a good baseline model that works on a broad range of optimization problems. Nonetheless, the choice of even more suited parameters and design space in the EA can make stabilization of the optimum both quicker and more reliable (Rahnamayan *et al.* 2007; Kazimipour *et al.* 2014). As these choices are highly dependent on the optimization problem at hand, no automatic adaption of the parameters is included. Hence, the following sections is intended to offering intuition on when and whether to deviate from the presented default choices and in which direction.

Regarding the initial population size (step 1), the goal should be to obtain good coverage of the initial search space (Zaharie and Micota 2017). Therefore, with more parameters or larger search intervals, it can make sense to increase the size of the initial set of parameter settings to a couple of thousand. In our example, even scenario 1 could have easily been improved in terms of required computations by not considering cases of more than 250 test bulls in the initialization step. In case simulations require a high computational load (Pelikan *et al.* 2000; Piszcz and Soule 2006), it might be necessary to reduce the initial set of parameter settings. However, one should be aware that this will increase the risk of running into a local maximum (Bajer *et al.* 2016).

To assess parameter settings more effectively, it may be advantageous to evaluate scenarios with multiple replicates within a single iteration (step 2). This approach is particularly relevant for extremely small breeding programs and short time horizons where stochasticity can be a major factor in the evaluation. However, even for our small minimal example, such extensive replication was not necessary. Replication can also be employed as a strategy to estimate the variability of the outcome of the simulation and then be integrated into the objective function to provide a more robust and accurate optimization solution.

In our approach, replication is achieved indirectly through kernel regression, which efficiently aggregates results by averaging similar settings. This relies on the fact that parameter settings with comparable values can be treated as replicates, as small differences between them do not significantly affect outcomes. Additionally, we implement a repeat mechanism for the best-performing parameter settings. The performance of these settings from previous iterations is re-evaluated in subsequent iterations using different random seeds, thus accounting for stochastic variability.

For selecting the best parameter settings (step 3), we recommend selecting more parameter settings in the first couple of iterations, as there is still more diversity present and to avoid the loss of potentially promising settings. In our example, we selected 100 parameter settings in the first two iterations, 50 parameter settings in iterations 3–9, and 30 parameter settings afterward with similar splits between steps 3.1, 3.2, and 3.3 (Table 1).

For generating new parameter settings (step 4), the same number of new parameter settings (300) in each iteration is generated. However, as step 4.3 is mostly intended for fine-tuning already promising settings, this is not applied in the first few iterations, and more focus is given to steps 4.1 and 4.2.

To avoid running into local optima, one could for example extend the generation of new parameter settings in this step by randomly sampling parameter settings similar to the initialization. Note that most of the simulations generated this way will be highly explorative with a low likelihood of providing good solutions. Hence, greatly increasing computational load which at least in this relatively simple breeding scheme was not deemed necessary by us.

### Practical considerations in optimization of breeding programs

In some instances, even if the value of the objective function remains relatively stable, there might be variation in individual parameter settings across iterations (Moscato 1989). This has practical implications in real-world scenarios, and it enables breeders to potentially allocate resources differently or achieve the same outcome through alternative scenarios, which might be logistically more feasible. In this regard, the definition of a suitable target function is of major importance, as from practical experience defining such an objective function is practically not easy to put all inputs and outcomes of the breeding program in a general equation. Here, visual manual human assessment can also help to check if the suggested optimum from the EA is not only in the defined search space but also in the realm of solutions a breeder would grant reasonable/doable.

For practical breeding, it would also be conceivable to run the EA framework multiple times, potentially with different target functions to then perform an in-depth analysis to calculate key characteristics (genetic gain, inbreeding, etc.) from these optima and pick the preferred solution. By this, the abstract concept of a target function can be replaced with a practical choice between which combination of genetic gain/inbreeding is the most desirable. Different target functions could for example be generated by using different weightings of genetic diversity and short-/long-term genetic gain.

### Computational setup for optimizing a specific breeding program

In the context of economic considerations for optimization strategies, a crucial aspect involves evaluating the costs against potential benefits. For our minimal example, with a cloud computing service charging around 0.012€ per CPU core hour and 0.012€ per 6 GB of memory/hour https://hpc.ut.ee/pricing/calculate-costs, the total per-job cost, combining core hours and RAM usage, is 0.55 cent per simulations. Running 40 iterations with 12.300 simulations would therefore result in a cost of 67.65€. In most commercial industrial practices, the focus of optimization lies in finding the best solution within a specific operating region or parameter space of interest that meets cost-effectiveness criteria and generates profits within a specified timeframe. In scenarios where a breeding program has limited prospects for improvement, allocating a substantial budget for optimization, may not be economically justified, particularly as the required computing time for larger breeding program simulation will be substantially higher.

### Termination criteria

Although the use of a fixed termination criterion and assessment of the state of the framework is desirable for computational pipelines, we strongly recommend also relying on manual human assessment, at least in support, given the time-consuming nature of simulating real-world breeding programs. Visual inspection of the change in target function and individual parameters is a common practice with evolutionary algorithms (Almeida *et al.* 2015). In many cases, fine-tuning the parameters associated with termination criteria relies often on an iterative, trial-and-error approach (Jain *et al.* 2001).

To define general termination criteria, we propose to assess the optima from all previous iterations using a kernel regression of all simulations. If users observe no substantial changes or improvements over successive iterations in both parameter settings and the objective function’s value but also the computational cost arising from the algorithm, they may consider stopping the optimization process earlier. The user can also specify a threshold for improvements if the results over ten iterations fall below a certain level. This threshold needs to be chosen depending on the specific optimization problem and is highly dependent on the desired precision of results (Jain *et al.* 2001; Ghoreishi *et al.* 2017).

A possible alternative approach could be, to only run a small number of iterations and increase it if the need arises after further evaluation. By integrating Snakemake into our EA framework, we enhanced the flexibility in determining when to stop the algorithm without the need to rerun initial iterations. This is possible because a Snakemake process can be paused and then re-evaluated, deciding independently which steps need to be rerun, run additionally, or which results can be reused.

## Conclusion

In conclusion, our study presents an innovative optimization framework using an EA that integrates a local search approach based on a kernel regression model. Our framework shows superior optimization efficiency to existing approaches and applies to both classes and continuous variables, hereby, enabling breeders to explore a wider range of scenarios compared to traditional methods (Bančič *et al.* 2024). The results across all problems indicate that our proposed framework shows great promise by robustly estimated optima while significantly reducing computation time. This study demonstrates that the EA algorithm consistently converges towards a common optimal solution, showcasing its robustness and ability to identify globally optimal or near-optimal configurations. The algorithm’s superior speed in terms of how quickly it stabilizes the optima, along with its solution diversity, balance between exploitation and exploration, and robustness to stochasticity, highlights its potential for larger breeding optimization tasks. The adaptable nature of our proposed framework makes it not only suitable for various future projects but also ensures flexibility in accommodating different breeding program designs. Users can easily modify, extend, or replace steps and adjust parameter choices as necessary. Thus, our framework supports optimization strategies that adjust to changing needs in breeding programs.

## Data Availability

Supplementary Files S1 and S2 have been shared with the scientific community at Figshare: https://doi.org/10.6084/m9.figshare.27231909.v4. The presented evolutionary framework is patent pending under application numbers EP24164947.4 and EP24188636.5. Patent applicants are BASF Agricultural Solutions Seed US LLC and Georg-August-Universitaet Goettingen. Inventors are Torsten Pook, Azadeh Hassanpour, Johannes Geibel, and Antje Rohde. Academic, noncommercial use is possible under a public license with all examples supported with scripts on GitHub repository details given at: https://github.com/AHassanpour88/Evolutionary_Snakemake/blob/main/License.md.
